# In silico simulation of liver crack detection using ultrasonic shear wave imaging

**DOI:** 10.1186/s12880-018-0249-5

**Published:** 2018-05-16

**Authors:** Erwei Nie, Jiao Yu, Debaditya Dutta, Yanying Zhu

**Affiliations:** 1 College of Information and Control Engineering, Liaoning Shihua University, Fushun, People’s Republic of China; 2 College of Science, Liaoning Shihua University, Fushun, People’s Republic of China; 3 Sensing and Computer Vision, Union Pacific Railroad, Omaha, USA

**Keywords:** Ultrasonic shear wave imaging, Liver crack, Speckle tracking, Directional filter, Edge detection

## Abstract

**Background:**

Liver trauma is an important source of morbidity and mortality worldwide. A timely detection and precise evaluation of traumatic liver injury and the bleeding site is necessary. There is a need to develop better imaging modalities of hepatic injuries to increase the sensitivity of ultrasonic imaging techniques for sites of hemorrhage caused by cracks. In this study, we conduct an in silico simulation of liver crack detection and delineation using an ultrasonic shear wave imaging (USWI) based method.

**Methods:**

We simulate the generation and propagation of the shear wave in a liver tissue medium having a crack using COMSOL. Ultrasound radio frequency (RF) signal synthesis and the two-dimensional speckle tracking algorithm are applied to simulate USWI in a medium with randomly distributed scatterers. Crack detection is performed using the directional filter and the edge detection algorithm rather than the conventional inversion algorithm. Cracks with varied sizes and locations are studied with our method and the crack localization results are compared with the given crack.

**Results:**

Our pilot simulation study shows that, by using USWI combined with a directional filter cum edge detection technique, the near-end edge of the crack can be detected in all the three cracks that we studied. The detection errors are within 5%. For a crack of 1.6 mm thickness, little shear wave can pass through it and the far-end edge of the crack cannot be detected. The detected crack lengths using USWI are all slightly shorter than the actual crack length. The robustness of our method in detecting a straight crack, a curved crack and a subtle crack of 0.5 mm thickness is demonstrated.

**Conclusions:**

In this paper, we simulate the use of a USWI based method for the detection and delineation of the crack in liver. The in silico simulation helps to improve understanding and interpretation of USWI measurements in a physical scattered liver medium with a crack. This pilot study provides a basis for improved insights in future crack detection studies in a tissue phantom or liver.

## Background

The liver is one of the most commonly injured organs in abdominal trauma [1] and liver trauma is responsible for significant mortality [2]. A timely detection and precise evaluation of hepatic injury and bleeding site is necessary because the injury, if undetected, may progress to a more severe state, and even be life-threatening. Imaging hepatic injured site (a.k.a. laceration or crack) with conventional B-mode ultrasound may be difficult [3–5] because the injured area usually appears hypo-echoic on the sonogram due to hemorrhage. Unlike tumor masses, which have good contrast resulting from highly heterogeneous echotexture, cracks in homogeneous soft tissues lack the necessary contrast. It becomes notoriously difficult to detect when the crack in the liver is juxtaposed. There is a need to develop better imaging modalities for hepatic injuries to be integrated into a bedside ultrasound imaging system to increase the sensitivity of ultrasonic imaging techniques for sites of hemorrhage caused by cracks. This paper aims to present a method for detecting the liver crack (or bleeding site) using an ultrasonic shear wave imaging (USWI) based technique.

USWI has been found to be a valuable noninvasive tool for studying the elastic properties of biological tissue. It relies on accurate estimates of tissue motion between frame-to-frame deformations of the tissue. For USWI, the shear wave is generated by pushing the tissue remotely with an ultrasound transducer, and with the same transducer, the tissue deformation during shear wave propagation is recorded in real-time ultrasonic images [6, 7]. Usually afterwards, the shear wave post-processing in shear wave elasticity imaging uses inversion algorithms to reconstruct shear modulus and acquire the tissue elasticity distribution.

In this study, we conduct an in silico simulation of the ultrasonic shear wave imaging of the crack in a liver tissue model by using the ultrasound RF signal synthesis and speckle tracking technique. Different from the post-processing for the shear wave elasticity imaging, the crack localization in this study is implemented by applying a directional filter and edge detection algorithm rather than recovering the elasticity map. We will discuss the crack detection with our method. To the best of our knowledge, study using shear wave imaging based method for liver trauma has been rarely reported and very little is known about the usefulness of USWI for an improved identification of hepatic injured site. The current study is performed to improve understanding and interpretation of USWI measurements in a scattered liver medium with a crack.

## Methods

### Finite element simulation

Assuming liver is a purely elastic solid, a two-dimensional (2-D) homogeneous and isotropic tissue medium (5.0 × 5.0 cm^2^) was constructed using a finite element (FE) package (COMSOL). Structural Mechanics Module was used for this study. At the center of the liver medium, a rectangular (3.8 cm × 0.05 cm) excitation rod was created, and a uniform plane shear wave was produced by oscillating the rod vertically with one cycle of a 100 Hz low frequency harmonic vibration. As the shear wave propagated sideways away from the center, the tissue medium was displaced along the axial direction.

**Fig. 1:**
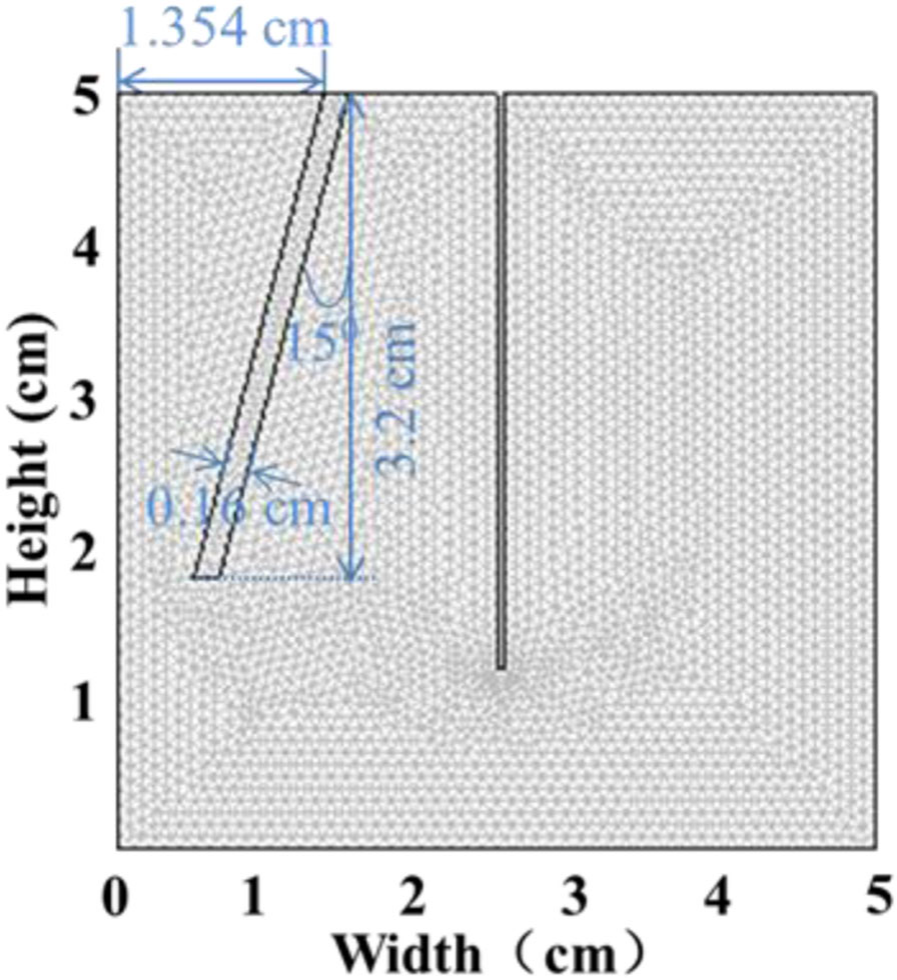
Schematic diagram of FE mesh of the simulation model

The liver medium with the following characteristics was simulated: density *ρ*_1_=1.2 × 10^3^ kg/m^3^, Poisson’s ratio *v*_1_=0.499, and Young’s modulus *E*_1_=6 kPa [8, 9]. The Young’s modulus value corresponds to a shear elasticity of about 2 kPa, which is appropriate for the liver, based on the earlier literature [10–12]. The crack was 3.2 cm in depth, inclined downward from the upper surface at an angle of 15 degrees from the vertical direction. The crack was located between *x* = 1.354 cm and *x* = 1.519 cm at the top, with a uniform thickness of 1.6 mm (horizontal thickness was 1.65 mm). The medium between the two edges within the crack had a density of *ρ*_0_=1.06 × 10^3^ kg/m^3^, Poisson’s ratio of *v*_0_=0.499, and Young’s modulus of *E*_0_=0.005 Pa, to mimic the blood [13]. The boundary conditions were assumed to be: free at the top surface of the liver tissue and the boundary of the excitation rod; fixed at the bottom surface boundary of the liver tissue; roller at the other surfaces (left, right boundaries) and the crack boundary [9, 14]. A mapped mesh with triangular elements was employed (altogether, 26,808 elements). Figure 1 shows the schematic diagram of the FE mesh of the simulation model. A time-dependent analysis was performed. With a frame rate acquisition of 5000 frames/s, the simulation was run from 0 ms to 21.0 ms with a time step of 0.2 ms, over 106 frames. Spatial and temporal profiles of propagating shear waves were recorded. The recorded time-varying axial displacements were subsequently processed in MATLAB (MathWorks Inc., USA). To avoid the influence of the boundaries and reduce unnecessary computations, only data located in the region of interest, with the *X* coordinate between 0 and 25 mm and *Y* coordinate between 0 and 50 mm, were exported and processed. The output axial displacement data is a three-dimensional matrix (200 × 400 × 106), which indicates the data containing 106 frames and divided into 200 and 400 units in the *X* and *Y* directions, respectively.

### Ultrasound RF signal simulation

**Fig. 2:**
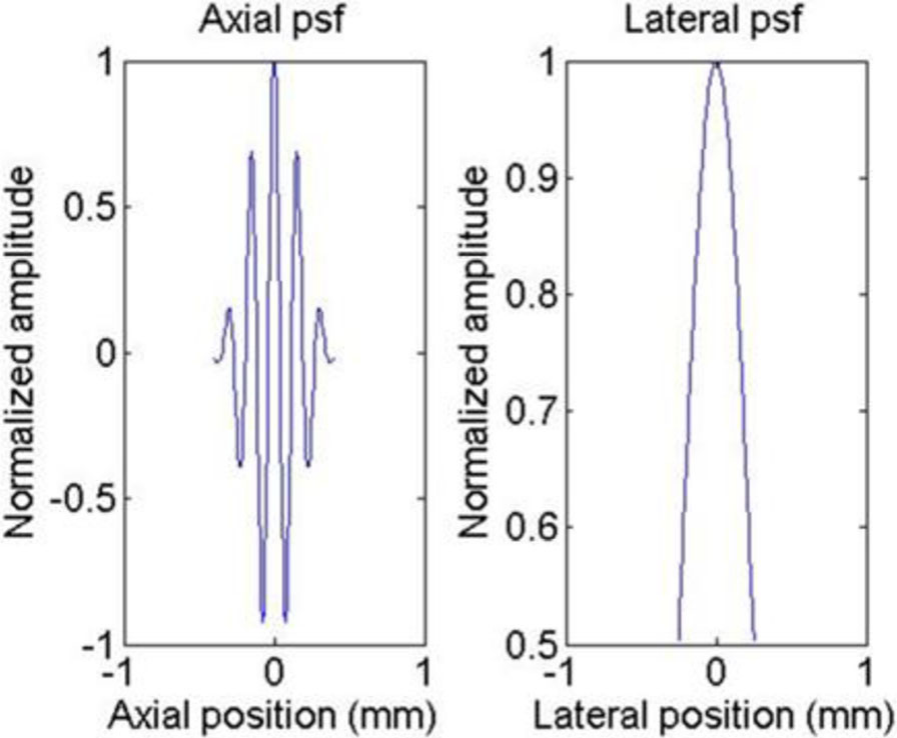
The axial and lateral components of the transducer PSF

The ultrasonic A-lines of the tissue are generated using a 2-D linear scattering model to simulate the actual ultrasonic shear wave imaging [15]. The transducer center frequency is set at 5 MHz, with the axial component of the transducer point spread function (PSF) having a 50% half-power relative bandwidth and the lateral component of the PSF having a full width at half maximum of 0.5 mm [16]. Figure 2 shows the axial and lateral components of the transducer PSF.

Each frame of digital RF data was then produced with a 40 MHz sampling frequency by convolving the 2-D PSF with the ultrasound scattering function. The scattering function was defined as the random distribution of ultrasound scatterers over the entire tissue area. For each time step of the FE simulation, the displacement of each scatterer was interpolated from the nodal solution and added to the pre-deformed coordinates to obtain the deformed scattering function. The speed of sound in soft tissue was assumed to be 1540 m/s. The scatter density was set as six scatterers per wavelength [14, 17]. Each RF image with a size of 5.0 cm × 2.5 cm (corresponding to 2596 axial samples × 256 beams) was generated at each time step of the deformation [9, 18]. The ratio of the mean envelope amplitude over the standard deviation in the B-scan image gives a value of 2.3, which means that the simulation produced fully developed speckle [19].

### Phase-sensitive 2-D speckle tracking

After ultrasonic RF signal synthesis, a phase-sensitive correlation-based 2-D speckle tracking algorithm was applied to the RF data to estimate the displacement between the frames [20]. Frame-to-frame axial and lateral displacements were estimated from the position of the maximum correlation coefficient from the cross-correlation on the baseband complex signals derived from the RF data.

The ultrasound speckle size was estimated to be 0.270 mm in the axial direction and 0.586 mm in the lateral direction from the 2-D correlation function of the baseband signals. The search kernel of the speckle tracking was set to be about the ultrasound speckle size for optimal displacement estimation with minimum variance [20]. Axial displacements were then refined using the phase zero-crossing of the complex correlation functions. To enhance the signal-to-noise ratio with reasonable spatial resolution, the adjacent correlation functions were filtered using 0.781 mm (lateral) by 0.308 mm (axial) separable Hanning filter. The search region was 0.781 mm in both dimensions. The frame-to-frame axial displacements were then accumulated over the entire 105 frames reference to the original geometry, to estimate the total displacement [21]. With the accumulation of frames, the displacement SNR was enhanced by reducing any uncorrelated errors in the axial displacement estimates [20].

### Crack localization without reconstructing the elasticity map

Unlike shear wave elastography, which applies the conventional local inversion technique for mapping tissue elasticity, the crack localization in this study is implemented by applying a directional filter and image processing without the conventional elasticity reconstruction.The axial displacement data, from 2-D speckle tracking after the ultrasound RF signal simulation, and directly from the FE simulation, are both processed using the directional filter. The directional filter was previously used [22–24] in the pre-processing of shear modulus reconstruction to suppress the artefact in the shear velocity estimation caused by the reflected shear wave. In the present work, the reflected shear wave becomes a “signal” rather than a “noise.” The directional filter decomposes the plane shear wave propagation in the [*k*,*w*] domain by performing a fast Fourier transform on the axial displacement data at all depths and then extracting the first and third quadrants, followed by the second and fourth quadrants, separately, to conduct the inverse fast Fourier transform [24]. In this way, the original shear wave was separated into its incident and reflected shear wave parts. By computing the absolute value of the displacement for each frame in the reflected wave and then accumulating over the entire imaging period, the total reflected wave field amplitude was estimated. Finally, the edge detection algorithm (Sobel method) was applied to the accumulated reflected wave magnitude image to find the crack. The detected crack locations, using speckle tracking and FE simulation, are compared to the given crack location and the relative errors are evaluated.

### Detection of cracks with different sizes and contours

Two supplementary studies were carried out investigating detection of cracks with different sizes and contours. In one study, the crack was reduced to 0.5 cm in depth and 0.5 mm in thickness, with the shape and direction unchanged. The crack was located between x = 1.467 cm and x = 1.519 cm at the top, with the right edge remained immovable but shortened. In another study, we considered a crack with a curved edge. The crack was made from a segment of the intersection of two circles. The two circles are of the same radius (4.5 cm), with centers located at the same height (5 cm) but different horizontal positions (x = − 3.146 cm, x = − 2.981 cm). The crack was still located between *x* = 1.354 cm and *x* = 1.519 cm at the top, but 1.8 cm in height. Following the same procedure as described above, the slimmer crack and the curved crack were studied for crack detection to gain an understanding of the robustness of our method.

## Results

**Fig. 3:**
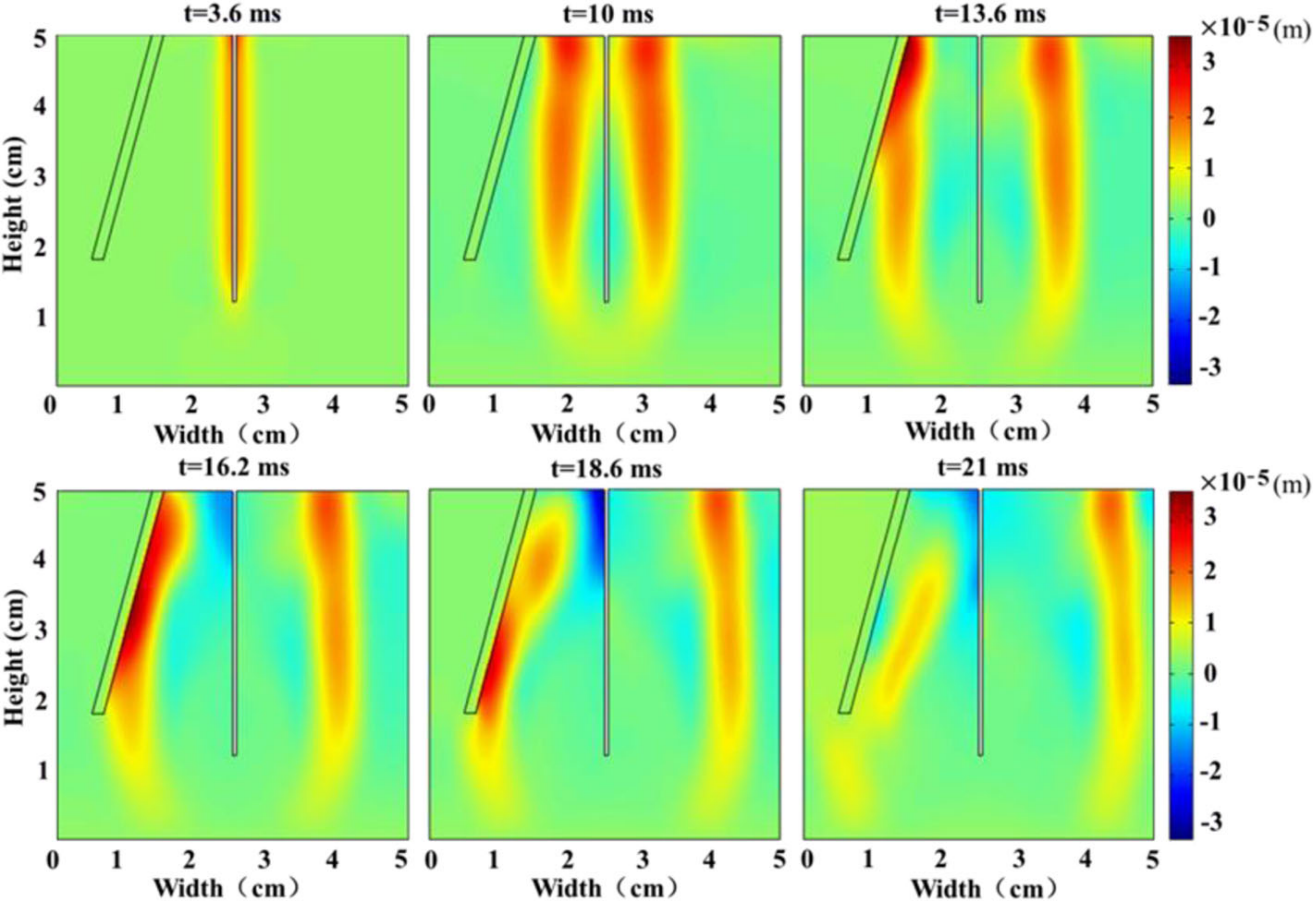
The axial displacement images during the shear wave propagation in the liver-mimicking medium

Figure 3 shows the axial displacement images during the shear wave propagation in the liver-mimicking medium. The shear wave speed observed from the movie is about 1.3 m/s, which is consistent with the expected value from the known Young’s modulus of 6 kPa for a purely elastic medium ($c=\sqrt{E/\left(3\rho \right)}$), thus, the wavelength is 1.3 cm. From Fig. 3, we can see that the shear wave is largely reflected when arriving at the crack, with a minimal portion passing though the crack and continuing to travel forward, despite the fact that the crack thickness is much smaller than (about 1/10 of) the shear wavelength. Subject to the fluid–solid interface influence (Scholte wave) [25], the waveform close to the top of the images is slightly deformed.

**Fig. 4:**
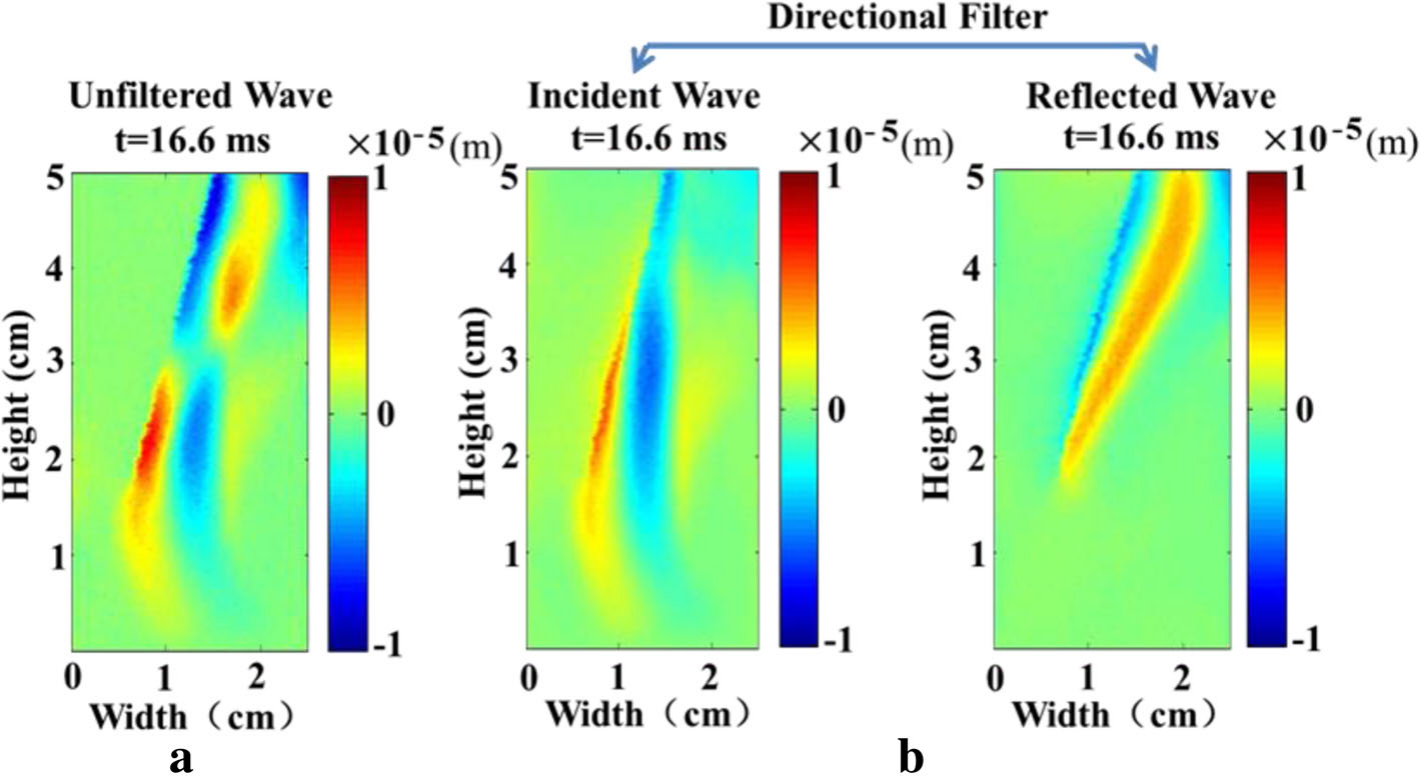
The use of the directional filtering algorithm for the displacement data obtained from speckle tracking at 16.6 ms. **a** A snapshot of the unfiltered waveform (at 16.6 ms). **b** Snapshots of the incident and reflected waveforms (at 16.6 ms), separated by the directional filter

Figure 4 shows the use of the directional filtering algorithm for the displacement data obtained from speckle tracking at 16.6 ms. The use of the directional filter (Fig. 4b) separates the incident and reflected waveforms (at 16.6 ms), in comparison with the unfiltered wave (at 16.6 ms) in Fig. 4a, which makes the observation of the shear wave more intuitive.

**Fig. 5:**
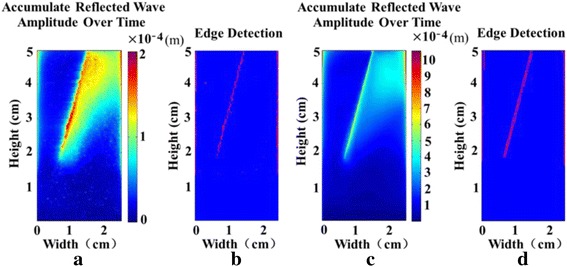
The crack localization after the use of the directional filter. **a** Accumulated reflected wave amplitude over time for imaging using speckle tracking. **b** Crack localization result for imaging using speckle tracking. **c** Accumulated reflected wave amplitude over time using FE simulation. **d** Crack localization result using FE simulation

Figure 5 shows the crack localization after the use of the directional filter. Figure 5a, c give the accumulated reflected wave amplitude over the entire 105 and 106 frames for imaging using speckle tracking and FE simulation, respectively. Compared with the FE simulation, the displacement data obtained using the speckle tracking method is underestimated. Figure 5b, d display the crack localization result after the use of the edge detection algorithm on the accumulated reflected wave amplitude image in Fig. 5a, c. Two oblique lines with equal length are detected in Fig. 5d. By referring to Fig. 5c, we know that the oblique line on the left in Fig. 5d is the boundary, while the oblique on the right in Fig. 5d is the artefact caused by the reflection of the shear wave. The detected oblique line on the left corresponds to the right edge of the given crack, and the left edge is not detected. In Fig. 5b, the detected crack edge is in the form of point distributions. Despite being discontinuous, the contour can still be visualized and matches generally well with the result in Fig. 5d. The detected crack depth is 3.09 cm and 3.08 cm in Fig. 5b, d, respectively, in comparison with the given depth of 3.2 cm.

**Fig. 6:**
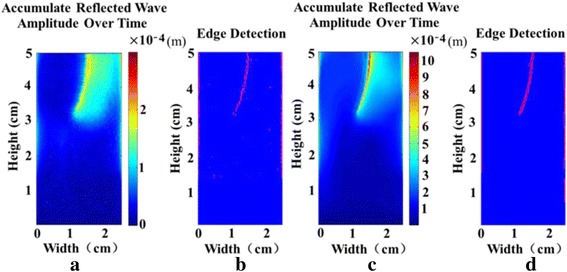
The crack localization for the curved crack. **a** Accumulated reflected wave amplitude over time for imaging using speckle tracking. **b** Crack localization result for imaging using speckle tracking. **c** Accumulated reflected wave amplitude over time using FE simulation. **d** Crack localization result using FE simulation

**Fig. 7:**
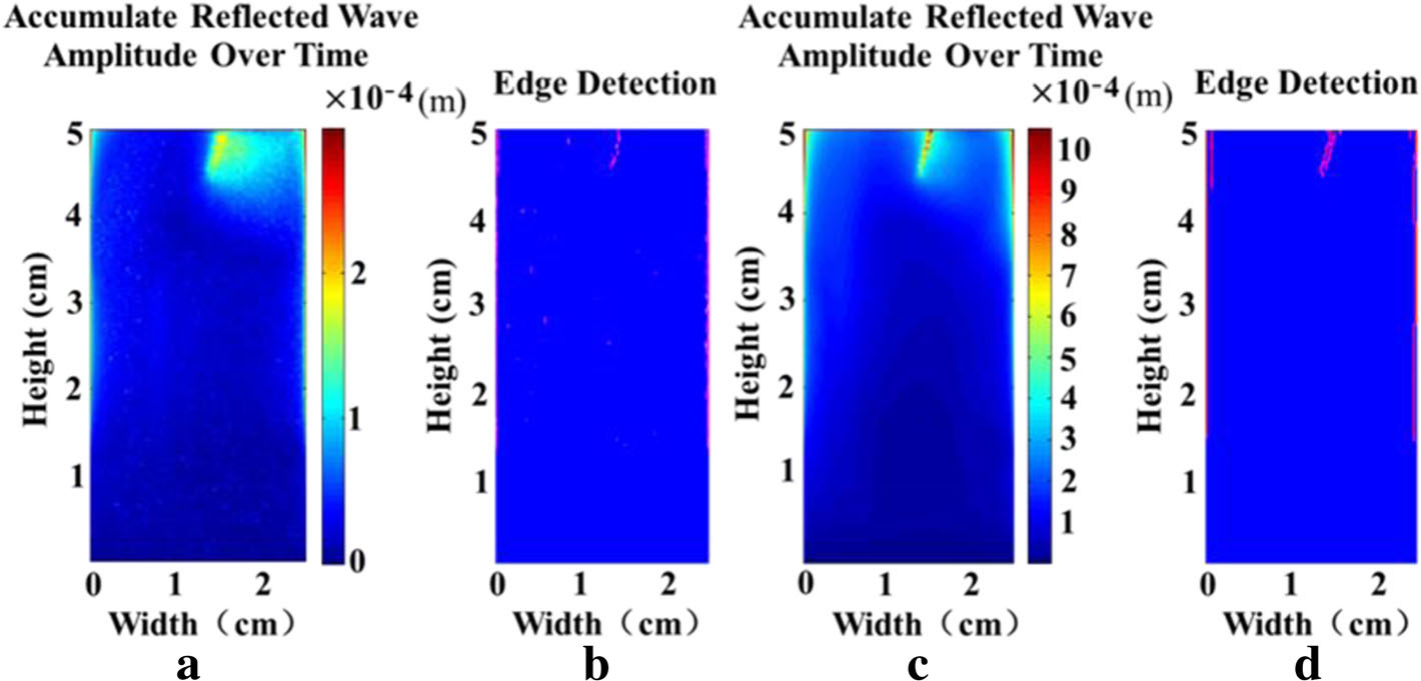
The crack localization for the slimmer crack. **a** Accumulated reflected wave amplitude over time for imaging using speckle tracking. **b** Crack localization result for imaging using speckle tracking. **c** Accumulated reflected wave amplitude over time using FE simulation. **d** Crack localization result using FE simulation

Figures 6 and 7 present the crack localization for the curved crack and slimmer crack, respectively, by using the same method as in Fig. 5. In both figures, the crack is well detected and localized using USWI. The detected crack depth is 1.79 cm and 0.45 cm for the curved crack and slimmer crack, in comparison with the given depth of 1.8 cm and 0.5 cm, respectively. The slimmer crack, located close to the upper surface, gives the largest error, 0.5 mm, in the depth estimation.

**Fig. 8:**
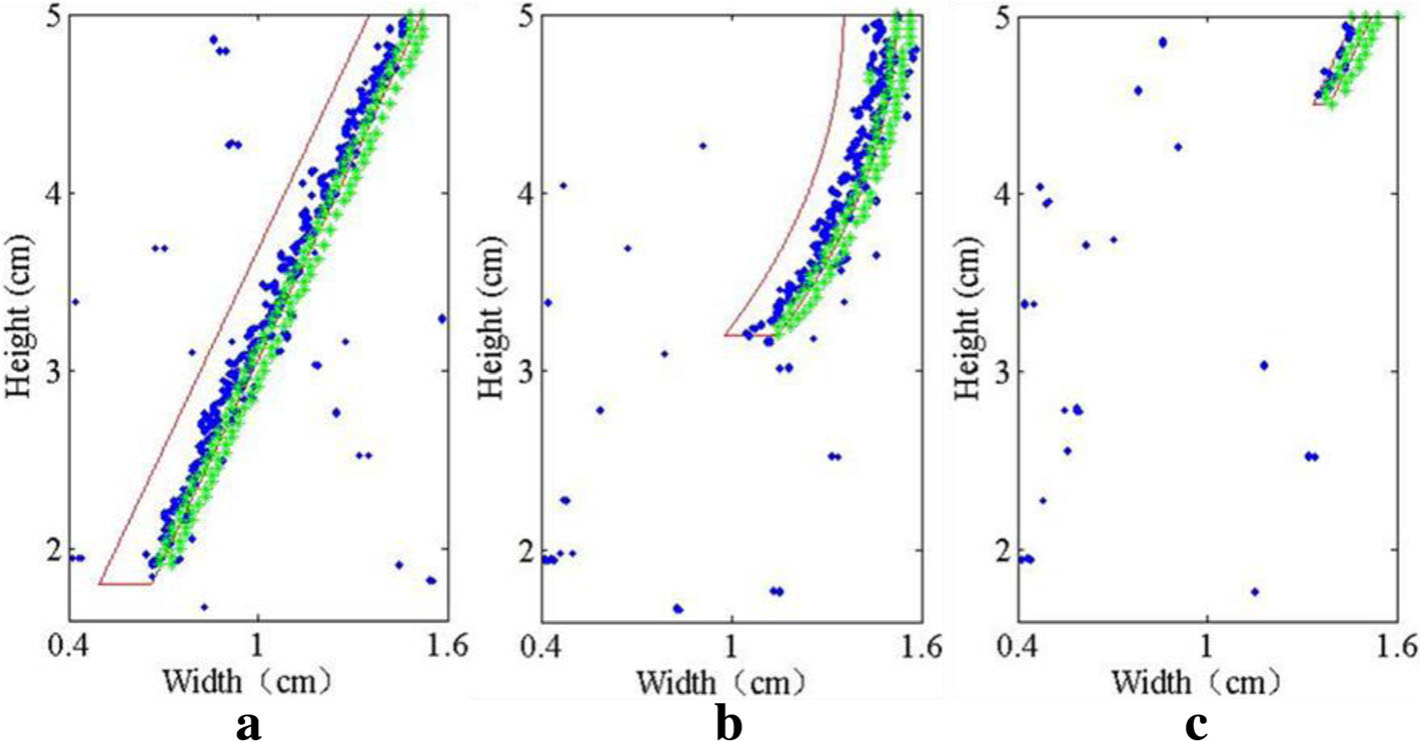
Comparisons of the detected crack locations using speckle tracking and FE simulation, with the given crack location. The contour of the given crack is represented by red lines. The detection results using speckle tracking and FE simulation are represented by blue and green scatter plots. **a** Straight crack (3.2 cm in depth). **b** Curved crack (1.8 cm in depth). **c** Slimmer crack (0.5 cm in depth)

In Fig. 8, the detected crack locations are compared using speckle tracking and FE simulation, with the given crack location. The contour of the given crack is represented by red lines. The detection results using speckle tracking and FE simulation are represented by blue and green scatter plots. From Fig. 8a, b, it can be seen that for a crack of 1.6 mm thickness, straight or curved, the right edge of the given crack is detected with our method, while the left edge is not detected. The most accurate detection of the right edge is from the FE simulation (the green scatter plot on the left) which is just next to the given right edge and on its left side. The use of speckle tracking moves the detected position further to the left. This is true for Fig. 8c as well, with the only difference that the crack is so slim (0.5 mm thick), that the left edge is also nearby in Fig. 8c. Out of the given crack, sparsely distributed artefacts can be observed in the simulation of the actual USWI by applying speckle tracking. Tissue is less homogeneous in the scattering model, and there is a tradeoff between the resolution and smoothness of the image. Generally a larger filter would reduce such artefacts, but the resolution would also be reduced.

Table 1 presents the quantitative comparison of the transverse positions of the detected and given right edges of different cracks at different depths. In Table 1, USWI represents the simulation result of USWI using speckle tracking. The relative errors of the detected midpoint positions in comparison with the given positions are listed. Except for the depth very near the upper surface, the detected locations are all a bit to the left, in comparison with the given locations, but the relative errors are all within 5%. The slimmer crack and the straight crack share the same right edge location, and the detected locations are identical too, except for very near the upper surface where the upper surface has an effect on the detection.

**Table 1:** The quantitative comparisons of the transverse positions of the detected and given right edges of different cracks at different depths

Depth (cm)	Straight crack (transverse position, cm)			Curved crack (transverse position, cm)			Slimmer crack (transverse position, cm)		
	Given	USWI	Error	Given	USWI	Error	Given	USWI	Error
0.208	1.464	1.431	−2.25%	1.515	1.525	0.66%	1.464	1.422	−2.87%
0.291	1.441	1.431	−0.69%	1.510	1.461	−3.25%	1.441	1.431	−0.69%
0.416	1.408	1.373	−2.49%	1.500	1.476	−1.60%	1.408	1.373	−2.49%
0.917	1.274	1.216	−4.55%	1.425	1.373	−3.65%	–	–	–
1.291	1.173	1.128	−3.84%	1.328	1.265	−4.74%	–	–	–
1.709	1.062	1.039	−2.17%	1.183	1.137	−3.89%	–	–	–
2.083	0.961	0.922	−4.06%	–	–	–	–	–	–
2.500	0.850	0.833	−2.00%	–	–	–	–	–	–
2.917	0.738	0.726	−1.63%	–	–	–	–	–	–

## Discussion

Liver trauma, particularly blunt liver trauma, is an important source of morbidity and mortality worldwide. Liver trauma may be induced by road traffic crashes, fall, antisocial, violent behavior, or concussive impacts during military operation. In current emergency medical care, diagnostic peritoneal lavage (DPL) or Focused Assessment with Sonography for Trauma (FAST) is used to diagnose hemoperitoneum in unstable patients with abdominal trauma [26]. The focus of DPL or FAST is to check for free intraperitoneal blood [26–29]. DPL is sensitive, but it is an invasive procedure. FAST is noninvasive and highly specific, but has a relatively limited sensitivity (72 and 46% in detecting blunt and penetrating abdominal trauma, respectively [3]). A negative FAST result does not exclude significant intra-peritoneal bleeding [26] or hepatic injury [3]. Unlike DPL or FAST, computed tomography can determine the source of hemorrhage [4]. CT examination provides superior images of traumatic pathology and is the golden standard for detecting liver injuries. However, it requires patient transport from the emergency department and more time to acquire images which limits its use for unstable patients. There is a critical need to develop supplemental diagnostic tool for liver trauma that can rapidly localize and evaluate traumatic liver injuries to be used in clinical situations where CT is not suitable or not easily accessible and FAST is insufficient in characterizing the site of hemorrhage, e.g., pregnant patients and children, patients requiring an emergent bedside procedure, patients under observation or during recovery period requiring serial abdominal examinations, and patients at a community hospital before being transferred to a trauma center, etc.

Since A. P. Sarvazyan, S. Y. Emelianov, et al. [30, 31] proposed the shear wave elasticity imaging method, ultrasound shear wave elasticity imaging has experienced rapid development and has successfully been used in breast lesion detection [32] and liver fibrosis staging [33]. In recent years, with the development of liver trauma research, studies have been carried applying ultrasonic shear wave elastography for liver trauma evaluation [34, 35]. Shear wave elastography was used to diagnose acute liver trauma by measuring the elasticity value of normal liver tissue and traumatic lesions created with a hemostat [35] or to assess the effects of local tissue repair in blunt hepatic trauma after haemostatic injection by measuring the elasticity value of treatment area at different time points [34]. However, to the best of our knowledge, there have been no reports on the application of ultrasound shear wave elasticity imaging (or ultrasonic shear wave elastography) or other USWI based method for the detection and localization of the crack (a.k.a. laceration) in liver, one of the most often ruptured organs in traumatic abdominal injuries.

Ultrasound shear wave elasticity imaging (or ultrasonic shear wave elastography) uses local inversion techniques for measuring tissue elasticity. Typically, these inversion techniques obtain the elasticities by estimating the phase speed from the gradient of the phase or an algebraic inversion of the elastic wave equation [36]. In estimating phase speed of the shear wave, a very good signal-to-noise ratio (SNR) is required [37]. In the wave equation inversion, the assumption of tissue homogeneity is violated at tissue interfaces, where shear wave reflection at structural interfaces may lead to incorrect speed estimates [38, 39]. Besides, calculation of the spatial-temporal derivatives of the displacements in the inversion algorithm also requires very low noise [40]. The use of USWI combined with a directional filter based technique in this study is motivated by the above perspectives. We expect our method, without elasticity map reconstruction, to be relatively more robust against noise and suitable for delineation of boundary. It is also expected to be computationally faster. Our method, if proved to possess these merits in future studies, even through technical improvements, could potentially be very useful for the in vivo applications. In this paper, we aim to present this method and conduct a preliminary study simulating its use for the detection and delineation of the liver crack.

The generation and propagation of the shear wave in a liver medium with a crack was simulated using a FE method. Numerical simulation suggests that the presence of the crack largely affects the propagation of the shear wave, and reflection of the shear wave at the crack is clearly observed. Despite the crack thickness to be nearly 1/10 of the shear wavelength, little shear wave can pass through the crack and propagate on the other side of the crack. This explains in part why only the near-end edge of the 1.6 mm thick crack is detected in the study. Under such circumstance, in order to detect the far-end boundary, one may move the ultrasound probe to the other side of the crack. As the crack becomes even thinner, a larger proportion of the shear wave will be transmitted through the crack, and the far-end edge of the crack may be detected. However, it may not be necessary to show whether the far-end edge of the crack is detected or not, because, as shown in Fig. 8c for the slimmer crack, as long as the near-end edge is detected, the far-end edge should be just by its left side.

To simulate the USWI method for the detection and localization of the liver crack in silico, the ultrasound RF signal synthesis and 2-D speckle tracking algorithm are used. In this study, the 2-D PSF used to synthesize the 2-D ultrasound RF signal closely mimics a typical commercially available linear probe. The configurations and algorithms for RF signal synthesis and ultrasonic tracking of displaced scatterers are designed to best exploit the shear wave phenomena for better detection and delineation of the crack in liver tissue. 2-D correlation-based speckle tracking is a widely used technique for elasticity imaging studies [20, 41–43]. Different from the techniques based on the signal amplitude which require numerical interpolations, the speckle tracking method used in this study is phase-sensitive. In the literature [44], it was suggested that a small weighted correlation kernel and correlation filter can be used to reduce displacement variance while retaining high spatial resolution. Therefore, for the tissue displacement estimation, we apply correlation kernels that are of the order of the autocorrelation width of the ultrasound signal, along with correlation functions filtering to suppress the errors in the displacement estimation and produce high spatial resolution.

It was observed that the accumulated reflected wave amplitude in Fig. 5a is less than that in Fig. 5c. We surmise that this discrepancy arises from underestimation of the tissue motion caused by correlation-based speckle tracking and resulting shearing under the PSF [45, 46]. The detected crack length using speckle tracking is 96.6% of the given crack length for the crack that is 3.2 cm in depth. We expect that the detected crack length is slightly shorter than the actual one, because the introduction of the scatterers results in a portion of the sound energy being scattered, which reduces the sensitivity of imaging. Increasing frequency may increase the sensitivity, but the image may become noisier.

Comparing the edge detection results using speckle tracking and FE simulation, the introduction of the speckle limits the accuracy of depicting the near-end boundary location, even for a fully developed speckle field. The detected locations using speckle tracking and FE simulation are on the left side of the given location of the crack’s right edge, and we think that it may be because there are still shear waves travelling through this edge into the crack which makes this interface not absolutely “hard” for wave reflection. In this study, the “Sobel” edge detection operator is used; if a more complex edge is to be detected, a more advanced contour extraction algorithm [47, 48] may be needed.

In terms of major findings, the curved crack and the slimmer crack are generally consistent with the straight crack. The contour of the curved crack can also be well delineated with the directional filter method. The slimmer crack that is 0.5 cm in depth and 0.5 mm thick can be detected too. Owing to its size and location that is close to the upper surface, larger errors are yielded in the height estimation. Relative errors of right edge detection for all the cracks are within 5%. Despite that our method proved the capability in capturing small structures like the 0.5 mm thick crack, more studies are needed to further suppress the artefacts in the background.For analyzing convenience, a plane wave model is used for the shear wave in this study. A more realistic simulation would generate a shear wave with a typical Mach cone-shaped acoustic radiation force impulse push. Nevertheless, the results obtained here indicate that although compared with an ideal homogeneous medium, variance in the displacement estimate using USWI in a scattering medium gives a larger error in the crack detection, USWI combined with directional filter based method seems to be a feasible and promising tool for localizing and depicting the liver crack.

As a pilot study, the circumstances considered here are that of the simplest case. Further studies will be necessary to understand the detection of cracks in more complex situations. It is reported that the sensitivity of FAST is higher (88–98%) for injuries of grade III or higher [3, 49]. Therefore, we focus on first studying our method for detecting hepatic lesions that are grade I through III which might be overlooked by FAST. We hope that our method will prove useful in the future, helping emergency clinicians and physicians detect subtle injuries or associated injuries that might be missed in the hepatic trauma setting. According to the American Association for the Surgery of Trauma (AAST), for liver trauma classification, laceration in grade I has capsular tear < 1 cm deep, laceration in grade II has capsular tear 1–3 cm deep, < 10 cm long, and laceration in grade III is > 3 cm deep. The straight crack, the curved crack, and the slimmer crack in this study should be classified as grade III, II, I, respectively. Despite that in clinical scenarios, size and location of the laceration do not have a necessary connection with the severity extent of the liver trauma, we feel that it is necessary to clarify the target of our model.

The three different cracks studied in this paper are all superficial lacerations, which indicate that there is capsular tear occurring for all three cases. As a preliminary study, hematoma is not considered in this paper. In fact, lacerations of liver trauma usually accompany perihepatic or subcapsular or intrahepatic hematoma, especially when the laceration is large. We will incorporate hematoma into our model and further investigate the robustness of our method in future work. Based on studies in Ref. [50], for imaging liver with ultrasound shear wave based method, the operator should verify that the region of interest (ROI) box is free of vascular structures. Properly placing the 2-D ROI box within liver parenchyma is critical for accurate detection of the crack, and it is important to make the size and position of the 2-D ROI box user adjustable. The reflection, the pulsatility, and the increased stiffness of the hepatic vessel wall will lead to measurement bias and variability.

Ref. [51] and Ref. [52] reported that in the clinical applications of shear wave elastography to liver stiffness assessment, ROI located 1–2 cm (or about 1.5–2 cm) below the liver capsule gives the most reliable result. In this paper, we observed the effect of the upper surface on the detection; however, because liver capsule is not modelled in the current study, the influence of the liver capsule on the detection of crack with our method is unknown. It would be interesting to incorporate the liver capsule and other possible interfaces (hepatic veins, two cracks, etc.) into our model and study their effects on the propagation patterns of shear waves and crack detection. It is important to constantly reduce problems that affect quality of imaging and detection, so that a wide exploitation of ultrasound shear wave based techniques will be enabled for new diagnostic applications such as in liver trauma. A multi-directional filter seems to be particularly effective in handling propagation in multiple directions, and different techniques [53, 54] may help improve shear wave motion detection and image processing for better extraction of the crack. It would be also interesting to develop abilities to identify, quantify, and stratify traumatic damage to the liver by area and severity. Technical methods for orientation analysis and damage severity sorting based on automated edge detection [55] are potentially useful for hepatic crack evaluation.

Our future work will also involve in vitro and ex vivo studies for liver crack detection with USWI combined with the directional filter based method. The ultimate goal is to develop USWI based techniques to be a preferred tool in detecting traumatic hepatic injury and delineating the injured site, and use it for additional confirmatory evaluation in an Emergency room or as a supplemental diagnostic tool in the diagnosis of hepatic trauma to be implemented by a bedside ultrasound imaging system. Despite that the clinical feasibility is too early to be known and yet to be proven, the current study should move USWI based technique closer toward the goal for such applications.

## Conclusion

In this study, we conduct an in silico simulation of liver crack detection and delineation using ultrasonic shear wave imaging (USWI). The generation and propagation of a shear wave in a liver tissue medium having a crack is simulated using FE model. Ultrasound radio-frequency (RF) signal synthesis and a 2-D speckle tracking algorithm are then applied to simulate the actual USWI in a medium with randomly distributed scatterers. We present a method that applies a directional filter and edge detection algorithm solely without recovering the elasticity map for the detection of the crack, and with our method, the near-end edge of the crack can be well localized. For a crack with a thickness of 1.6 mm (about 1/10 of shear wavelength), we found that little shear wave can pass through it and as a result the far-end edge of the crack cannot be detected. The detected crack length using USWI is slightly shorter than the actual crack length. We test our method for cracks with varied sizes and locations, and the near end edge of the crack can be detected and delineated, with an error within 5%. The robustness of our method for the detection and localization of the crack is demonstrated in a curved crack and a subtle crack of 0.5 mm thickness. Despite the various limitations in this study, to the authors’ knowledge, no other study has yet been reported, which has attempted to detect and delineate a hepatic laceration in traumatic liver injuries by using ultrasound shear wave imaging based techniques. The in silico study presented in this paper provides a basis for more advanced crack detection studies in a tissue phantom or liver.

## Abbreviations

2-D: Two-dimensional
AAST: American Association for the Surgery of Trauma
CT: Computed tomography
DPL: Diagnostic peritoneal lavage
FAST: Focused assessment with sonography for trauma
FE: Finite element
PSF: Point spread function
RF: Radio-frequency
ROI: Region of interest
SNR: Signal-to-noise ratio
USWI: Ultrasonic shear wave imaging
